# Prepubertal Daidzein Exposure Enhances Mammary Gland Differentiation and Regulates the Expression of Estrogen Receptor-Alpha and Apoptotic Proteins

**DOI:** 10.5402/2011/896826

**Published:** 2011-09-04

**Authors:** Prachi Mishra, Anand Kar, R. K. Kale

**Affiliations:** ^1^School of Life Sciences, Jawaharlal Nehru University, New Mehrauli Road, New Delhi-110067, India; ^2^School of Life Sciences, Devi Ahilya University, Khandwa Road Complex, Indore-452 017, MP, India

## Abstract

Mechanism of chemoprevention by daidzein (500 **μ**g/g bwt) was examined by injecting it subcutaneously at 16th, 18th, and 20th day postpartum, followed by counting of terminal end buds (TEBs), terminal ducts (TDs), and lobules and immunohistochemistry of ER-**α**, Bcl2, Bax, and caspase-3. DNA fragmentation was also analysed to measure the apoptosis level. Estradiol benzoate (EB) (500 ng/g bwt) and dimethyl sulphoxide (DMSO) were used as reference and vehicle, respectively. Observations show a significant enhancement of mammary gland differentiation at postnatal day 21 (PND21) as well as PND50. There was a significant decrease of ER-**α** expression at PND21 and increase in its expression at PND50, in daidzein-treated animals. The ratio of expression of Bcl-2 to Bax proteins increased at PND50 the same whereas, it decreased at PND50 due to daidzein. An increased expression of caspase-3 and DNA fragmentation was also seen due to daidzein at PND50. The mammary gland of EB-treated animals showed response a somewhat similar to that of daidzein-treated animals.

## 1. Introduction

Phytoestrogens, naturally occurring hormone-like compounds with a unique diphenolic structure found in several plants [1], have received much attention as dietary components as they are known to promote better health. Phytoestrogens have been also associated with reduced incidence of various cancers, cardiovascular disease, and osteoporosis and lower cholesterol level [2–5]. Some studies suggest that the dietary intake of phytoestrogens decreases the risk of breast cancer in humans [6, 7]. The most extensively studied phytoestrogen in vitro and in vivo is genistein which is found richly in soybeans. However, biological activities of many other naturally occurring isoflavones have not been studied in detail till now. The phytoestrogen daidzein is an isoflavone present as a glucoside in many plants used in human diets. Daidzein is especially concentrated in soy and soy-based products used for human consumption [8, 9]. A higher incidence of breast cancer in Western population is seen as compared to Asians who consume diet containing relatively high levels of low-fat, high-fibre, high-soya content [10]. Asian women show protection against breast cancer in comparison to women living in USA or Britain [11, 12] which is believed to be lost upon immigration and exposure to Western lifestyles within a few generations [13]. These studies suggest that exposure to phytoestrogens at an early stage is extremely important, in order to gain their cancer-preventive effects. 

The female mammary gland undergoes cell division during puberty, and throughout adult life there is cyclical proliferation and involution during the estrous cycle. During early development, rising endogenous estrogen promotes mammary duct branching which ends in highly proliferative structures, terminal end buds (TEBs) [14]. At postnatal day (PND) 21, the number of TEBs is maximal, and at puberty the onset of estrous cycles results in changing progesterone and estrogen levels that promote differentiation of TEBs to the less proliferative alveolar buds or lobules [15–17]. Highest number of tumours per animal was observed when carcinogen exposure occurred in rats at postnatal day 40–50, a time when mammary gland exhibits a high density of the highly proliferative TEBs [15]. The incidence of carcinomas is positively correlated with the number of TEBs in mammary gland of the young virgin rat at the time of carcinogen exposure [16, 17]. Administration of the phytoestrogen genistein to neonatal and prepubertal rats reduced TEBs by promoting TEB differentiation [18, 19]. 

A study on Sprague Dawley rats (mammary tumor model) shows that daidzein has been an effective inhibitor of DMBA-induced mammary tumor [20]. Chemoprevention study from our lab has also shown a decrease in tumor burden and incidence by prepubertal daidzein administration [21]. Although daidzein also has the ability to bind to the ERs [22], the precise mechanism of action by which it shows a chemoprevention against breast cancer has not been clearly elucidated. Considering the above literature, it could be hypothesized that daidzein could also affect the differentiation and signaling pathways as a mechanism of chemoprevention. Hence, to explore the mechanism of actions of daidzein, its effect on cell proliferation, mammary gland differentiation, and the expression of ERs in a prepubertal mammary tumour model was carried out.

## 2. Materials and Methods

### 2.1. Chemicals

Daidzein, estradiol benzoate (EB), and Hoechst 33258 were bought from Sigma Aldrich Co. (St. Louis, Mo, USA), while dimethyl sulphoxide (DMSO) and carmine were bought from Qualigens (India). Primary antibodies for ER-*α*, Bcl2. Bax, and caspase-3 were bought from Santa Cruz Biotechnology, Inc., Santa Cruz, Calif, USA, and secondary antibodies, diaminobenzidine (DAB), avidin-biotin complex, and normal Goat Serum (NGS) were purchased from Bangalore Genei (India).

### 2.2. Animals

Female Sprague Dawley rats, kept in animal facility in a 12 hr day : 12 hr night cycle, and maintained in the animal house of the university were used for the present study. Standard feed and water were provided *ad libitum*. The studies were conducted according to the ethical guidelines of Committee for Control and Supervision of Experiments on Animals, Government of India, on the use of animals for scientific research.

### 2.3. Experimental Design

The whole investigation was carried out under two major experiments.

**Experiment 1**

A total of thirty-six animals (female Sprague Dawley offsprings) of the same age were divided into 3 groups of 12 each. On postnatal days 16, 18, and 20, while group II and III were injected subcutaneously with 500 *μ*g/g body weight daidzein and 500 ng/g body weight of estradiol benzoate (EB), respectively [19, 23], an equal volume of vehicle (DMSO) was injected to the animals of group I (Control, DMSO). On the 21st day, 18 hours after the last injection, 6 animals from each group were sacrificed and the rest of the 6 were maintained till the 50th day and sacrificed thereafter. The mammary glands obtained at both times (PND21 and PND50) were preserved for differentiation study.

**Experiment 2**

In another 36 animals, a similar experiment was repeated. However, this time the mammary glands obtained at PND21 and PND50 were stored for immuno-histochemical experiments.

### 2.4. Mammary Gland Differentiation

All mounts of fourth pair of the mammary gland of the animals were prepared. Mammary glands were dissected at the time of sacrifice, spread on the microscope slide, and then placed in neutral buffered formalin for 8 h (22-day-old animals) and overnight (50-day-old animals). Glands were defatted in acetone for 4 h or overnight, placed in 70% alcohol for 30 min, hydrated in water (15 min), and stained in alum carmine for overnight. After staining, glands were run through a series of graded alcohols (30–100% ethanol) and placed in xylene to clear the tissue. Glands were then compressed between two glass slides for 24 h, released and allowed to expand for at least 8 h, and finally mounted using a glass coverslip and DPX. All mounts were then evaluated via light microscopy using the criteria established by Russo and Russo [16, 17] and Murrill et al. [19]. All mounts were evaluated for the number of terminal end buds, terminal ducts, and lobules type I by the help of the software ImagePro [19]. 

### 2.5. Immunohistochemistry (ER-*α*, Bcl2, Bax, Caspase-3)

The mammary glands dissected were fixed in 10% neutral buffered saline, and sectioning was done by cryostat at 20 microns. Sections were treated with 10% normal goat serum (NGS) for 2 h for blocking and then incubated in the primary antibody for 3 days at 4°C. The antibody was diluted in 0.1 M PBS containing 5% NGS and 0.5% triton-X-100. After being washed in PBS, the sections were incubated in biotinylated goat antimouse IgG at 1 : 200 for 18 h at 4°C. Following this secondary antibody step, the sections were washed and placed in preformed avidin-biotin peroxidase complex (ABC) at 1 : 400 dilutions for two hours at room temperature. For visualizations of the reaction sites, the sections were treated with the chromogen diaminobenzidine (DAB) and hydrogen peroxide for 2-3 min. The sections were rinsed with distilled water dehydrate in ethanol and coverslipped with distyrene plasticizer xylene (DPX) mounting medium.

### 2.6. Nuclear Staining

The nuclei were stained with Hoechst 33258 (Sigma); 10 *μ*m sections were mounted on gelatin-coated slides and washed in PBS for 10 min (Figure 6). They were incubated with Hoechst 33258 (1 *μ*g/mL) for 30 min in dark at room temperature. After washing twice with PBS (5 min each) and once with distilled water (5 min), the slides were coverslipped with 10% glycerol and stored in dark. The slides were viewed in a fluorescent microscope under the blue filter (excitation wavelength = 343 nm, emission wavelength = 483 nm). Since Hoechst is membrane permeant, the procedure does not require membrane permeablization.

### 2.7. Analysis

Immunostaining was evaluated by examination of slides under a bright field microscope (Carl Zeiss Axioscop Mot 2) at magnification 400x, and images were captured through a digital camera for measurement of intensity and counting of cells. Intensities of immunostained cells were estimated by densitometry using morphometric software Scion Image. In order to count the number of ER, Bcl2, Bax, Caspase-3 positive cells and Hoechst stained apoptotic nuclei, magnified views (400x) of sections were captured. The total cells (unstained) and immunostained cells were counted with the help of the software ImageTool (UTHSCSA). Values were compared using one-way ANOVA (Sigma Stat, Jandel Scientific). 

## 3. Results

### 3.1. Mammary Gland Differentiation

**Figure 1:**
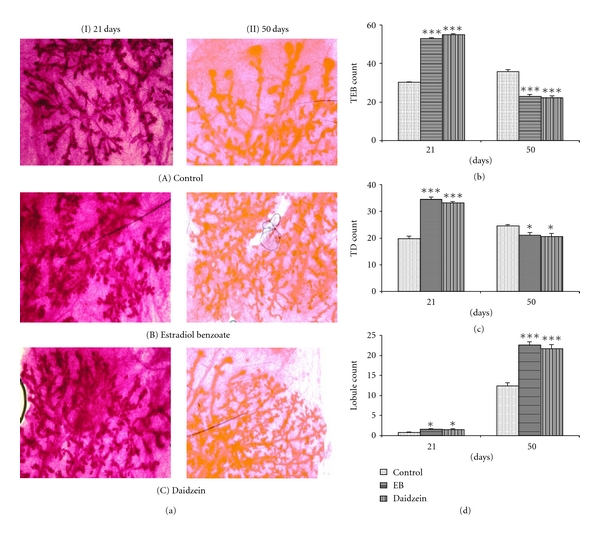
Effect of daidzein (500 *μ*g/g bwt) on differentiation of mammary gland through count of TEBs, TDs, and lobules, in prepubertal female Sprague Dawley rats at PND21 and PND50. (a) All mounts of mammary glands. Mean percent change ± SEM in the count of (b) TEBs, (c) TDs, and (d) lobules, from 6 animals. *P < 0.05, **P < 0.01, ***P < 0.001, compared to the respective value in control group.

Differentiation of mammary gland was studied by observing the branching of mammary gland and the terminal end buds and ducts and lobular count at two different stages (i.e., PND21 and PND50) after a prepubertal exposure of daidzein to the animals. An increase in the count of TEBs, TDs, and lobules has been seen in the daidzein-treated rat mammary glands at PND21, whereas there is a decrease in the TEB and TD count and further increase of lobule I count at PND50 as compared to the control, as shown in Figure 1. A similar significant increase of TEBs, TDs, and lobules has also been seen in the EB-treated animals at PND21, and a decrease is seen in TEBs and TDs and an increase in mammary gland lobules at PND50. 

### 3.2. Immunostaining of ER-*α*

Immunohistochemistry results at PND21 in daidzein-and EB-treated mammary glands showed that the percentage ER+ cell count decreased significantly, that is, 41.92 ± 1.0%, *P* < 0.001 and 39.21 ± 0.8%, *P* < 0.05, as compared to the control (45.7 ± 0.5%), whereas intensity of daidzein-and EB-treated epithelial cells decreased up to 80.9%, *P* < 0.001, and 82.8%, *P* < 0.001, respectively, as compared to the control (100%). At PND50, daidzein-and EB-treated mammary gland epithelial cells in both the count and intensity were shown to be increased significantly. The percentage count of ER+ cells increased to 39.2 ± 0.7%, *P* < 0.001, and 41.54 ± 0.7%, *P* < 0.001, in daidzein-and EB-treated epithelial cells respectively, from control (33.65 ± 0.57%), and intensity increased to 121.5%, *P* < 0.001, and 125.4%, *P* < 0.01, in daidzein-and EB-treated epithelial cells, respectively from control (100%) significantly.

### 3.3. Evaluation of Bcl2/Bax Ratio

In 21-days-old mammary glands, the Bcl2+ cell count and intensity were found to be increased significantly in daidzein-treated animals (40.26 ± 0.38%, *P* < 0.001; 111%, *P* < 0.01) as well as in the EB-treated animals (44.16 ± 1.02%, *P* < 0.001; 122.4%, *P* < 0.001) from control (35.9 ± 0.6%, 100%), whereas the cell count and intensity of Bax+ cells were decreased significantly in daidzein-treated animals (19.37 ± 0.22%, *P* < 0.05; 82.0%, *P* < 0.001) as well as in EB-treated animals (19.7 ± 0.68%, *P* < 0.05; 83.6%, *P* < 0.05) from control (23.61 ± 0.91%, 100%). In 50-days-old mammary glands, Bcl2+ cell count and intensity were decreased significantly in daidzein-treated animals (31.3 ± 0.64%, *P* < 0.001; 82.3%, *P* < 0.01) as well as in the EB-treated animals (29.11 ± 0.65%, *P* < 0.001; 79.5%, *P* < 0.01) from control (40.4 ± 0.65%, 100%). Simultaneously, the cell count and intensity of Bax+ cells were increased significantly in daidzein-treated animals (40.65 ± 0.67%, *P* < 0.001; 114%, *P* < 0.05) and in the EB-treated cells (39.0 ± 0.84%, *P* < 0.001; 116.3%, *P* < 0.01) from control (28.05 ± 0.37%, 100%). Bcl2/Bax ratio was found to be enhanced in mammary gland at PND21 but not at PND50.

### 3.4. Evaluation of Apoptosis

**Figure 2:**
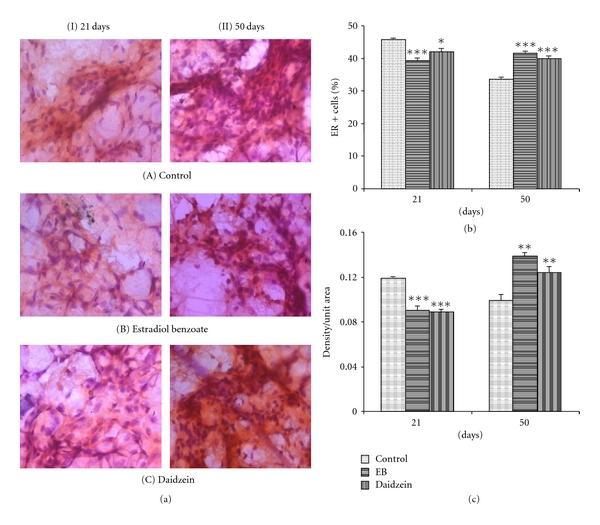
Effect of daidzein (500 *μ*g/g bwt) on ER-*α*. (a) Immunohistochemistry of ER-*α* in prepubertal female Sprague Dawley rats at PND21 and PND50. (b) mean percent change ± SEM of ER-*α*+ cell count and (c) mean ±  SEM of densitometric analysis, from 6 animals. *P < 0.05, **P < 0.01, ***P < 0.001, compared to the respective value in control group.

**Figure 3:**
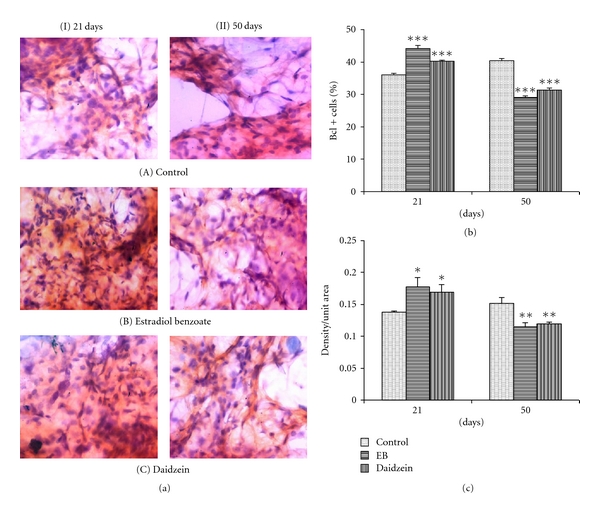
Effect of daidzein (500 *μ*g/g bwt) on Bcl2. (a) Immunohistochemistry of ER-*α* in prepubertal female Sprague Dawley rats at PND21 and PND50. (b) mean percent change ± SEM of ER-*α*+ cell count and (c) mean ± SEM of densitometric analysis, from 6 animals. *P < 0.05, **P < 0.01, ***P < 0.001, compared to the respective value in control group.

**Figure 4:**
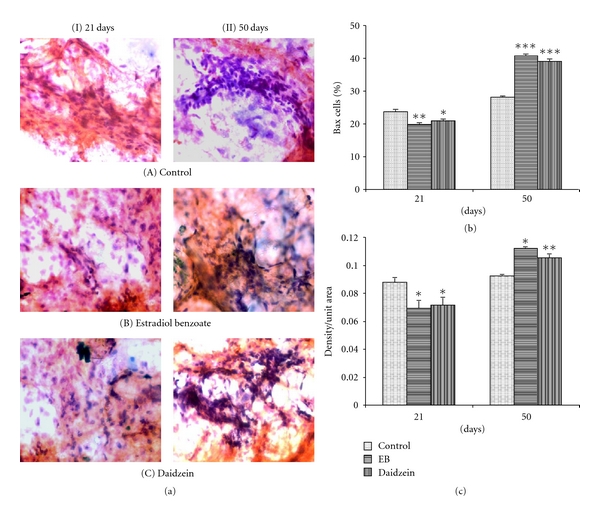
Effect of daidzein (500 *μ*g/g bwt) on Bax. (a) Immunohistochemistry of ER-*α* in prepubertal female Sprague Dawley rats at PND21 and PND50. (b) mean percent change ± SEM of ER-*α*+ cell count and (c) mean ± SEM of densitometric analysis, from 6 animals. *P < 0.05, **P < 0.01, ***P < 0.001, compared to the respective value in control group.

**Figure 5:**
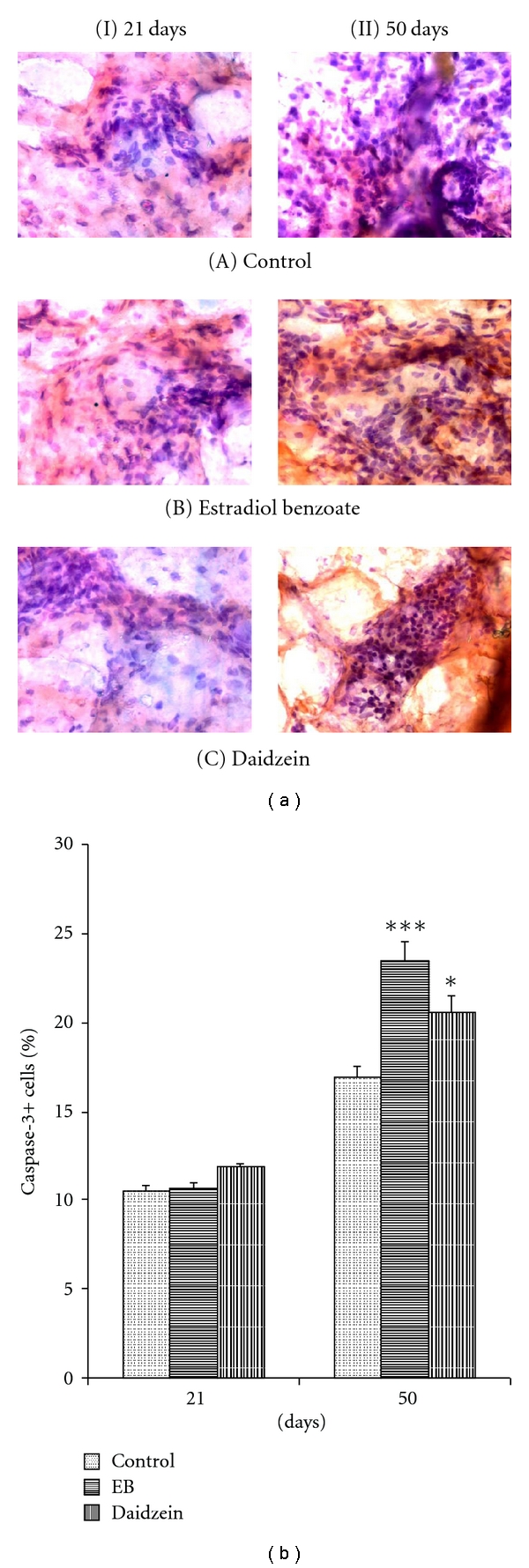
Effect of daidzein (500 *μ*g/g bwt) on caspase-3 count. (a) Immunohistochemistry of ER-*α* in prepubertal female Sprague Dawley rats at PND21 and PND50. (b) mean percent change ± SEM of Caspase-3+ cell count. Each bar represents the average ± SEM from 6 animals. *P < 0.05, **P < 0.01, ***P < 0.001, compared to the respective value in control group.

**Figure 6:**
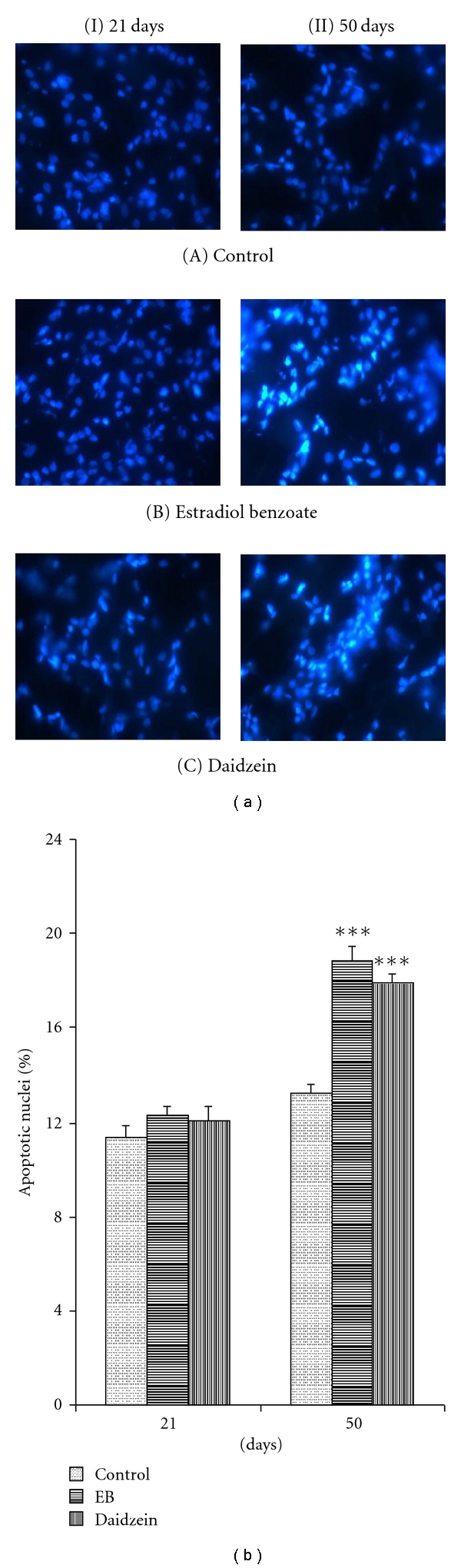
Effect of daidzein (500 *μ*g/g bwt) on level of apoptosis through Hoechst (33258) staining. (a) Pictomicrographs showing apoptotic nuclei in prepubertal female Sprague Dawley rats at PND21 and PND50. (b) mean percent change ± SEM of apoptotic nuclei count, in PND21 as well as PND50 mammary glands. Each bar represents the average ± SEM from 6 animals. *P < 0.05, **P < 0.01, ***P < 0.001, compared to the respective value in control group.

Caspase-3+ cell count percentage was shown to be increased significantly at 50 days in mammary glands treated with daidzein (20.57 ± 1.07%, *P* < 0.05) and EB (23.39 ± 1.21%, *P* < 0.001) as compared to the control mammary glands (16.92 ± 0.68%). Also the percentage apoptotic nuclei count increased in daidzein (17.94 ± 0.46%, *P* < 0.001) and EB (18.82 ± 0.71%, *P* < 0.001), compared to the control (13.28 ± 0.42%), at 50 days, significantly (Figures 2–5).

## 4. Discussion

Our data clearly demonstrates, the prepubertal exposure to daidzein by the female Sprague Dawley rats produces an enhancement in the differentiation of the mammary gland as well as influences apoptosis and the ER expression. The highest susceptibility of a rat mammary gland to a carcinogen occurs in the postpubertal virgin females around the age of 50 days, which correlates well with the higher number of TEBs and the high cell proliferation, and a low incidence of mammary carcinoma is related to the loss of TEBs and the low cell proliferation activity [24].

 There was an increase in the count of TEBs and TDs and lobules by the prepubertal administration of daidzein in PND21, which shows an increased proliferation in the cells, which is probably necessary for the developing of mammary gland at a prepubertal stage. Lobules are more stable structures than the TEBs and TDs, hence, by the increase in the count of lobules, it could be interpreted that the undifferentiated TEBs and TDs are progressing gradually towards the formation of more differentiated lobules, which could be due to the influence of daidzein. In a similar kind of study with genistein, it was observed that prepubertal genistein exposure increases the count of TEBs and lobules at PND21 [19, 23]. At PND50, the decrease in the count of TEBs and TDs indicated a decreased proliferation, and coupling this with an increase in count of lobules at the same time suggests further enhancement in the differentiation of mammary glands at pubertal stage, in daidzein-treated mammary glands. Earlier genistein had shown a similar potential to increase the mammary gland differentiation, along with reduction of TEB count and increase in the lobules, at PND50 [19, 23]. Some of the xenoestrogens such as diethylstilbestrol (DES), o,p′-DDT, Aroclor 1221, Aroclor 1254, and 2,3,7,8-tetrachlorodibenzo-p-dioxin (TCDD) also showed an enhancement in the mammary gland differentiation when administered prepubertally [25]. Since the development of mammary gland is controlled by female reproductive hormones like estrogen, it is likely that exogenously the administration of the estrogen agonist estradiol benzoate (EB) will show an enhancement in the mammary gland development, which has also been reported earlier [26]. Here, the EB-treated mammary glands also showed an increase in the TEBs and TDs at PND21 and a decrease in the TEBs and TDs count along with the increase in lobules at the old mammary gland PND50. A similarity in the results of both EB-and daidzein-treated mammary gland differentiation data suggests that daidzein might have a similar mode and magnitude of action as that of EB, when administered to rats during the prepubertal stage. Thus, the overall results of the mammary differentiation data reveals that shortly after exposure to daidzein, there was a rapid development of the mammary gland, yielding more differentiated structures (lobules) and fewer undifferentiated structures (terminal end buds).

Tumorigenesis experiments done earlier in our laboratory had also shown a decrease in tumour incidence and burden by treatment of daidzein to SD rats in the prepubertal stage [21]. Hence this chemopreventive effect of daidzein can be correlated to the increased differentiation rate of mammary gland caused by the prepubertal exposure of compound. 

ER-*α* plays a large role in mammary gland development. Immunohistochemical data of ER-*α* indicated that there is a decreased expression of ER-*α* in response to Daidzein, seen in the mammary epithelial cells at PND21. Normally, the percentage of ER-*α*-positive nuclei varies according to the developmental state of the mammary gland. Decreased expression of ER-*α* may also reflect proliferation, as proliferating epithelial cells of the mammary glands of young virgin rats do not express the receptor [27, 28], while its increase after 50 days suggests that ER-*α* expression might not be somewhat necessary for proliferation but may be required for the differentiation of mammary glands. This is consistent with our previous results which indicated that prepubertal daidzein treatment resulted in a proliferative mammary gland at PND21, which led to a more differentiated structure evident at PND50. A similar result in the expression of ER- *α* in response to daidzein and EB may suggest a common molecular mechanism of action.

There has been a link established between the proliferating epithelial cells and the expression of ER-*α* in developing rodent mammary gland. The increase in the count and intensity of Bcl2 at PND21 further supported the result that there might be an increase in proliferation of cells which might have resulted in the increased count of the unstable proliferating structure, TEBs, accordingly. Later at PND50, there is a decrease in the Bcl2 expression which indicates decreased proliferation. This can be related to the enhanced differentiation of the mammary glands in the daidzein-treated rats, where there was an increase in the lobule count along with a decrease in the count of TEB at PND50. And since the differentiation of the cells has increased and proliferation has slowed down, the count of Bcl2 has also decreased in 50-day-old daidzein-treated rats, significantly, as compared to control. Furthermore, a decrease in the level of antiapoptotic protein Bax at PND21 and its increase in PND50 show that apoptosis is not seen in PND21 rather it increased in PND50 as evaluated by the simultaneous expression levels of Bcl2 and Bax. The process of apoptosis is integral to normal mammary gland development [28], and the morphogenesis of ducts in the development of mammary gland is dependent on the selective death of epithelial cells to form mammary acini [29, 30].

Nuclear condensation and fragmentation because of degradation of DNA into oligonucleosome fragments [31], which is a characterized feature of apoptosis, was seen to be increased in PND50, whereas no significant change in the apoptotic level was observed at PND21. This was further confirmed when the apoptotic marker protein, caspase-3, which is also known to be one of the principal intracellular effectors of the apoptotic cascade, simultaneously increased in the daidzein-and EB-treated cells along with the increase in apoptotic nuclei, at PND50. 

Since daidzein and EB decrease the expression of ER-*α* in prepubertal epithelial cells along with the increase in the proliferation and differentiation of cells at the same time, it can be said that the estrogen receptor expression and the mechanism for the proliferation of cells may not be directly correlated, and both proteins might have been influenced by the test compound by separate pathways. Both processes may be independent of each other, but both might be required for the enhancement of differentiation of the gland.

## 5. Conclusion

The phytoestrogen daidzein has a potential to regulate the differentiation of mammary gland by maintaining a balance between proliferation and apoptotic death of the cells which is critical for its normal development. Perturbations in this balance can contribute to the development of various disorders in mammary gland including cancer. Conditions that upregulate cell proliferation or downregulate apoptosis can allow the accumulation of mutations that contribute to the subsequent development of breast cancer. Daidzein enhances the differentiation of mammary gland in a controlled manner at all the developmental stages. Although the influence of daidzein on some key proliferative and apoptotic proteins has been discussed here, investigations on other cellular pathways integral to the differentiation process can be explored to have a better understanding of the mechanism of actions of daidzein. Thus, it can be concluded that daidzein, in a similar mechanism of action as that of EB, influences the differentiation of mammary gland by affecting cell proliferation proteins and the ER-*α* expression. Since the more differentiated the mammary gland is, the less susceptible it is to cancer incidence, dietary intake of daidzein from an early developmental stages may be beneficial to women folk in particular, which can be further confirmed through clinical trials.
